# Gastritis in Children

**Published:** 1890-01

**Authors:** J. L. Cunningham

**Affiliations:** Fort Worth, Texas


### Correspondence.

### GASTRITIS IN CHILDREN—REPLY TO DR. WILSON'S LETTER.

FORT WORTH, TEXAS, December 21, 1889.

*Editor Daniel's Texas Medical Journal:*

In the last (December) issue of your journal, page 222, Dr. T. M. Wilson, says: "My baby boy, seven months and seven days old, died yesterday morning of acute gastritis, after an illness of thirteen days. * * * I lost my first child at four months and eleven days old, with gastro-intestinal catarrh. Lost our third, a female, aged two months and twenty-seven days, with acute gastritis. If you have anything special to give in such cases, please let we know; I am getting very anxious about the matter."

In your reply, you say: "We regret exceedingly to be unable to throw any light on the subject. Gastro-intestinal catarrh, or the summer disease of children, that condition which usually attends teething, is one of the opprobria of medicine; there is no specific." And you add, "We invite a discussion of the subject, throwing open our pages to those who, unlike Dr. Wilson, have had success in the management of the trouble. and we beg to suggest, no more practically important subject could well be presented for discussion. Let us hear from you, gentlemen."

I deeply sympathize with Dr. Wilson, and I entirely agree with you, that "no more practically important subject could well be presented for discussion."

Apart from the overwhelming sorrow of the parents and friends, what a loss to the commonwealth! The State is clamorous for immigrants to come and settle up the country. Every inducement is held out to Northern men, foreigners—anybody—

everybody—to come and take possession. But this same State sits quietly, and with a complacency perfectly appalling, sees, year after year, her innocents slaughtered by hundreds and thousands, in a way to make Herod blush, by that insatiable Moloch, cholera infantum. These little darlings are the hope of the country, and are, or should be, very precious to the State; and something ought to be done to stay the surging tide that annually sweeps so many of them from the shores of time.

As this is pre-eminently an age of money getting, and money loving and money worshipping, it would be well to view the subject from a money, or financial standpoint. What has the State lost in money by the disasters in Dr. Wilson's family? These children are surely worth as much to the State as negro children used to be worth to their masters. Eight hundred to one thousand dollars was the average price for a negro child in slavery times.

Three children, at the lowest amount, eight hundred dollars, would be twenty-four hundred dollars. Now, how many children die annually in Texas from cholera infantum and its congeners, gastritis and gastro-enteritis? Let us say five hundred—and the number is doubtless double this number—500x800=400,000. Five hundred innocents slaughtered annually, and nearly half a million dollars worse than thrown into the Gulf of Mexico. It would be well to remember that this wreck and ruin is the work of only one Moloch. Those other fiends of typhoid fever, diphtheria, etc., also get in their baleful work regularly, without any let or hindrance from the State. It is true, she says that the State Health Officer shall be "pledged to sanitation, as well as quarantine;" but every body knows that the State Health Officer utterly ignores and repudiates that part of the enactment relating to sanitation; and that the great State of Texas has, in fact, no agent to look after the sanitary affairs of the people, to instruct them how to avoid sickness, and to enforce hygienic and sanitary regulations so as to prevent preventable diseases.

*After* a person is bitten by a rabid animal, the auspicious moment for effective action is past. *After* a man is mortally wounded with the terrible six-shooter, the most skillful surgeon finds that all his remedies for shot wounds avail nothing. Prevention is better than cure. A grain of the former is worth a ton of the latter. Hereafter "prevention" must be the watchword. It will not do to charge the deaths of these children to "an inscrutable dispensation of Providence," or to say that it was inevitable (fatalism), or that it was bad luck. Effects invariably follow causes. If the laws of health were thoroughly studied and mastered, and carefully and systematically carried out, we would not hear so much of infants dying of acute "gastritis," and "gastro-intestinal catarrh."

Dr. Wilson's children were evidently poisoned by their food, which contained tyrotoxicon, or some other equally poisonous ptomain, the result of bacterian fermentation or putrefaction. It is scarcely probable that teething had anything to do with the trouble, as one of the children was only two months old, and the other only four.*

Dr. Wilson says nothing about the food (a most potent factor in causation) upon which the children were subsisted, but I infer they were all nourished from the same breast. It is a well known fact that breast milk is sometimes a veritable poison to the child, induced by certain states of the mother's mind, as violent anger, grief, etc.; by certain articles taken into her stomach, as medicines or food; or by certain physiological or pathological conditions incident to her sexual life, viz.: menstruation and gestation. The milk from the mother's breast is sometimes found, upon a careful analysis, totally unfit for use as a baby's food. Whenever the albuminoids are much above one or two per cent., or the fats greatly less than three or four per cent., there is sure to be trouble with the child.

The chemical quality of the milk is also influenced by the intervals of nursing. Too frequent nursing—say every hour, as when the child is fretful—will produce a concentrated milk by increasing the solids at the expense of the water; while nursing at long intervals will render the milk too watery, and consequently not nourishing. Upon the very first manifestation of

*This we pointed out also.—ED.

trouble with the stomach or bowels of children under two years of age, and particularly in hot weather, attention should be promptly given to the food, whatever it may be. It should be immediately stopped, and something else substituted for a short time. If the trouble still continues, steps should be then taken to rid the stomach and bowels of the poison, by the use of cathartics, and if need be, a thorough washing out of the stomach and colon with large quantities of warm water, holding in solution a little carbonate of soda. Then antiseptics, such as salicylate of sodium, resorcin, or napthalin, will be in order, to prevent permeation or putrefaction. In fact, the case should be treated just as if a poison had been swallowed.

J. L. CUNNINGHAM, M. D.

